# Epidemiological Characteristics of Dengue Disease in Latin America and in the Caribbean: A Systematic Review of the Literature

**DOI:** 10.1155/2017/8045435

**Published:** 2017-03-14

**Authors:** Jaime Rafael Torres, Tomás Agustín Orduna, Maricela Piña-Pozas, Daniela Vázquez-Vega, Elsa Sarti

**Affiliations:** ^1^Instituto de Medicina Tropical, Universidad Central de Venezuela, Caracas, Venezuela; ^2^Unidad de Medicina Tropical y Medicina del Viajero, Hospital de Infecciosas F. J. Muñiz, Buenos Aires, Argentina; ^3^Departamento de Servicios de Información, Instituto Nacional de Salud Pública de México, Cuernavaca, MOR, Mexico; ^4^Departamento de Epidemiología, Sanofi Pasteur LATAM, Coyoacán, CDMX, Mexico

## Abstract

Dengue, an important mosquito-borne virus transmitted mainly by* Aedes aegypti*, is a major public health issue in Latin America and the Caribbean. National epidemiological surveillance systems, usually based on passive detection of symptomatic cases, while underestimating the true burden of dengue disease, can provide valuable insight into disease trends and excess reporting and potential outbreaks. We carried out a systematic review of the literature to characterize the recent epidemiology of dengue disease in Latin America and the English-speaking and Hispanic Caribbean Islands. We identified 530 articles, 60 of which met criteria for inclusion. In general, dengue seropositivity across the region was high and increased with age. All four virus serotypes were reported to circulate in the region. These observations varied considerably between and within countries and over time, potentially due to climatic factors (temperature, rainfall, and relative humidity) and their effect on mosquito densities and differences in socioeconomic factors. This review provides important insight into the major epidemiological characteristics of dengue in distinct regions of Latin America and the Caribbean, allowing gaps in current knowledge and future research needs to be identified.

## 1. Introduction

Dengue, a mosquito-borne virus transmitted mainly by* Aedes aegypti*, is a major public health issue in Latin America and the Caribbean [1]. Morbidity and mortality in this region have increased substantially in the last decade, from 400,519 cases and 92 deaths in 2000 [2] to 2,386,836 cases—representing a case incidence rate of 435 cases per 100,000 inhabitants—and 1,318 deaths in 2013 [3]. There are four dengue serotypes, DENV-1, DENV-2, DENV-3, and DENV-4 [4], all of which are capable of causing disease. Immunity to the infecting serotype is life-long; however, cross-protection against other serotypes is of limited duration, and perversely, severe dengue disease occurs more often with a second or subsequent infection with a different dengue virus serotype and carries a mortality rate of 1–20% [5].

Current programs for dengue prevention [6] and control support measures to promote adequate surveillance and control of mosquito transmission, with an ecosystem approach [7]. Although larval source reduction is an effective vector control strategy, many dengue endemic countries do not have routine control measures in place or only implement these during epidemics [8–10]. Furthermore, the limited resources are often reallocated to other competing needs once the mosquitos and the disease appear to be controlled, invariably allowing reinfestation to levels where epidemic transmission recurs [10].

National epidemiological surveillance systems, usually based on passive detection of symptomatic cases, underestimate the true burden of dengue disease [11]. Although not entirely satisfactory, these surveillance systems are usually sufficient to track disease trends and to detect excess reporting and outbreaks, helping to inform decisions about health service priorities. Data reported to the Pan American Health Organization (PAHO) and the World Health Organization (WHO) have shown all four dengue serotypes to circulate in Latin America and the Caribbean during the period 2000–2013 and revealed wide variation in the incidence of reported dengue cases and case fatality rate across the region (Tables 1 and 2) [12].

The objectives of this study were to characterize the recent epidemiology of dengue disease in Latin America and the English-speaking and Hispanic Caribbean Islands in terms of incidence and mortality rates, disease severity over time, age groups affected, circulating serotypes, and other risk factors associated with the disease and to identify gaps in epidemiologic knowledge as well as future research needs. Since the epidemiologic trends of dengue disease in Brazil, Colombia, and Mexico have recently been evaluated in systematic reviews [13–15], these three countries are not included in our review.

## 2. Methods

We undertook a systematic review of the literature in accordance with PRISMA guidelines. The protocol used was registered at PROSPERO International prospective register of systematic reviews (PROSPERO 2015: CRD42015024447; available from http://www.crd.york.ac.uk/PROSPERO/display_record.asp?ID=CRD42015024447). We used Medline (PubMed), Lilacs, SciELO, Redalyc, Artemis, and Cochrane databases to identify original studies published from 1 January 2000 through to 31 December 2013 on the epidemiology and public health impact of dengue in Latin America and the Caribbean. We assumed that this would be a sufficient duration to allow for an accurate overview of the recent trend in the evolution of dengue epidemiology in the region. However, we recognized that some articles published after 2000 would include information dating back to the 1990s or earlier. In these cases, we decided that this information would also be relevant for inclusion in this review.

Text terms relevant to dengue and epidemiology (morbidity and mortality) were combined with the names of the countries of interest. The search strategy included the following terms: dengue and epidemiology, combined with the names of the countries in Latin America and the English-speaking and Hispanic Caribbean Islands as well as “dengue fever” (FD/DF), “dengue hemorrhagic fever” (FHD/DHF) and/or “dengue shock syndrome” (SCD/SSD), “severe dengue” (with and without warning signs), and “severe dengue” according to the current classification of the WHO. In addition, we undertook a search of the gray literature to retrieve information from relevant sources such as the regional Ministries of Health, PAHO, and WHO for additional relevant articles. The search was restricted to English or Spanish language articles.

Duplicate articles were initially removed from the electronic searches before screening for relevance based on titles and abstracts (where available) using predefined inclusion criteria. Articles were included if they provided information on general epidemiologic indicators of dengue disease (incidence, attack rate, seroprevalence, mortality, and lethality), epidemic intensity (frequency of hospitalization and severity of the condition), populations most at risk, serotype information, geography of the disease, vector control measures, epidemiologic surveillance systems, case definition, laboratory capacity, and capacity to respond with vector control measures and included epidemiologic studies with no restriction on age, sex, or ethnicity. There was no restriction on the type of article for inclusion (case series, cross-sectional studies, cohort studies, environmental studies, cluster studies, case-control studies, expert opinions, and notes to the editor), except for single case reports and review articles (so as to avoid duplication of published data). Articles of studies undertaken in Brazil, Colombia, and Mexico were also excluded as these had already been subject for earlier comprehensive literature reviews.

The selection of articles for inclusion was undertaken by a literature review committee (JRT, TAO, MPP, ES, and SBL). The literature search was extended by reviewing the reference list of all relevant articles identified for additional studies not captured by the electronic search. Summary tables were constructed that included an internal identification of the article, first author, citation details, the database where the item was obtained, country, region, or city where the study was conducted, age and gender of the participants, number of dengue cases (DF and/or DHF), type of evidence where data was obtained (clinical study, case reports, etc.), study design, date(s) the study was undertaken, identified serotypes, and method used for their identification. No attempt was made to contact authors for further clarification or missing information.

The data were analyzed and synthesized into a narrative summary structured around the type of design, target population characteristics, epidemiological indicators of dengue disease (prevalence, incidence, seroprevalence, mortality, lethality, and outbreaks), circulating serotype, geography of the disease, and other associated risk factors. We did not undertake a meta-analysis because of the heterogeneity in the study designs and outcomes reported. For example, studies differed in the way they reported the incidence of dengue with some reporting the rate per number of individuals and others the rate per number of children or a given age group.

## 3. Results

### 3.1. Study Selection

**Figure 1:**
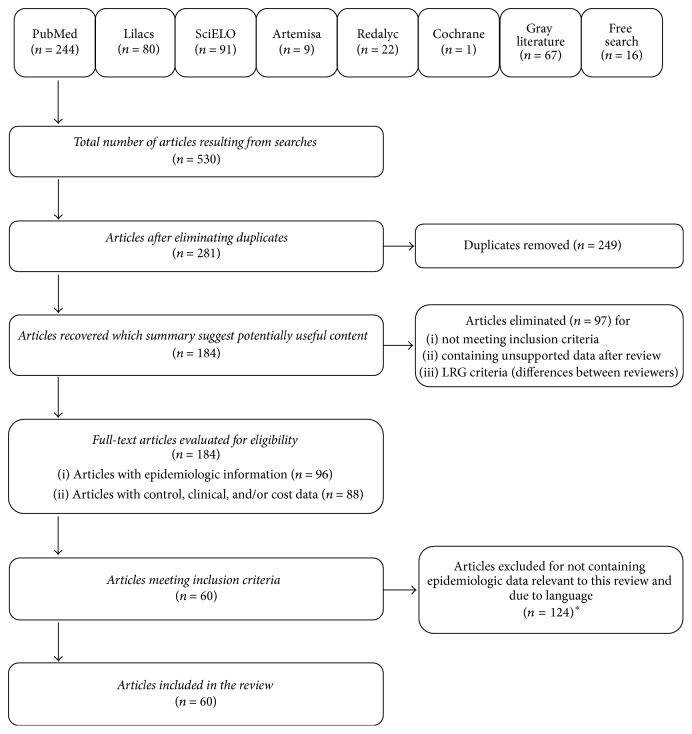
Flow diagram outlining the search strategy of the systematic review of dengue in Latin America, 2000–2013. ^*∗*^Articles eliminated because they did not contain any dengue-related data relevant to this review (*n* = 122) or were written in a language other than English or Spanish (*n* = 2).

The literature search identified 530 articles, 60 of which met our inclusion criteria (Figure 1): 21 case series studies, 19 cross-sectional studies, 10 cohort studies, six ecologic studies, one case-control study, one cluster study, and an expert opinion and note to the editor (Supplementary Table S1 in Supplementary Material available online at https://doi.org/10.1155/2017/8045435). The main epidemiologic characteristics from the studies included are described below by geographical region, including aspects related to outbreaks, seroprevalence, circulating serotypes, and morbidity and mortality rates. One previously published report on the epidemic patterns of dengue disease in the region of the Americas assessed cases reported to the PAHO between 1980 and 2007 [16].

### 3.2. Central America

Eighteen studies were identified for Central America and included data from Costa Rica [17–22]; Honduras [23, 24]; Nicaragua [25–32]; and Panama [33]. Specific data from the other countries in Central America (Belize, El Salvador, and Guatemala) were included as part of a wider review of dengue in the Americas [16]. DENV-2 circulated almost continuously from 2000 to 2008 (except for 2002) [18, 25, 28, 32] and was replaced by DENV-3 from 2008 to 2011 [32]. DENV-1 also circulated from 1999 to 2008 [21, 22, 24, 28, 30] and DENV-4 from 1999 to 2001 [25].

*Costa Rica* experienced peak dengue epidemics during 1997 [17] and 2003, with hospitalized patients having an average duration of hospital stay of 2.26 days for both epidemics [19]; overall, 10,308 dengue cases were reported between 1999 and 2004, with 2003 the year with the highest weekly incidence of reported cases [21]. Seroprevalence estimates in 206 asymptomatic children aged 1 to 10 years without a prior history of dengue during 2002-2003 ranged from 2.9% in inland areas (San José) of the country to 36.9% in the coastal zone (Puntarenas) [20]. The region of Pavas was identified as a local transmission zone for dengue virus between 2003 and 2007 [18]. In another study analyzing the cumulative incidence of DF/DHF from 1999 to 2007 across all 81 cantons (administrative divisions) in Costa Rica, the highest incidence of dengue was found mainly near the coast [22]. Temperature, altitude, and the human poverty index (HPI) were the main variables identified to explain the incidence of DF/DHF across these cantons.

In* Honduras*, the Metropolitan Health Regions of the Central District and San Pedro Sula contributed more than 45% of dengue cases registered in the country each year from 2007 to 2010; 64.4% of 1,692 cases reported across the country in 2007 were from these two health regions (overall, 2,128 confirmed cases were reported to PAHO that year, Table 1) [23]. The potential associations between climatic variation (during El Niño versus La Niña periods) and DHF (3,353 cases reported at the Hospital Escuela, Tegucigalpa) were assessed in another study in 2010 [24]. The La Niña phase was significantly associated with a higher incidence of DHF than the El Niño phase: there was a 158% difference in the mean incidence of cases reported during El Niño (−99% of cases below the mean incidence) to La Niña (+59% of cases above it) (*p* < 0.01). A lower Oceanic Niño Index (*p* = 0.0097), higher rain probability (*p* = 0.0149), accumulated rain (*p* = 0.0443), and higher relative humidity (*p* = 0.0292) were associated with higher DHF incidence.

During 2009-2010 in* Nicaragua*, there was an atypical presentation of dengue disease characterized by a significant increase in the number of patients with early symptoms of poor peripheral perfusion, that is, “compensated shock,” which resulted in an increase in the number of children transferred to intensive care compared with previous years in a cohort study (11.2% [19/170] versus 1.1% [2/181] cases) and hospital study (19.8% [42/212] versus 7.1% [16/225]) [29]. Among cases reported (*n* = 3,173) in Leon and Managua between 1999 and 2001 the highest burden of the disease was in children aged 5–9 years who accounted for 58% of all confirmed cases, but with the burden of severe disease predominantly in infants aged 4–9 months [25]. Secondary dengue infection was a risk factor for serious disease in children. In a 2-year cohort study of children aged 4–16 years conducted from May 2001 to May 2002 in Managua, the overall seroprevalence of DENV-specific antibodies in the children who remained in the entire study (*n* = 398) was reported to be 91%, with an increase from 75% at the age of 4 years to 100% by the age of 16 years [26].

The Pediatric Dengue Cohort Study, a community-based, prospective cohort study, initiated in Managua in 2004, is probably the most extensive study of the natural history and transmission of dengue in children in Nicaragua [27, 28, 30, 31]. The study initially recruited children aged 2–9 years (later extended to include those up to age 14 years) who were followed closely for all illnesses. In the period from August 2004 to March 2011, there were 448 symptomatic and 1,606 inapparent laboratory-confirmed DENV infections among 5,541 children who participated in the study over the years [27]. Both inapparent and symptomatic DENV infections were distributed equally among the sexes, but there was substantial variation in the proportion of symptomatic versus all DENV infections between study years. The mean age of infection was 1.2 years higher for symptomatic than inapparent DENV infections, and the duration of cross-protection induced by the first infection against a second symptomatic infection was estimated at 2 years. Interestingly, the incidence rate of secondary DENV infections (121.3 [95% CI: 102.7, 143.4] per 1,000 person-years) was significantly higher than for primary infections (78.8 [95% CI: 73.2, 84.9] per 1,000 person-years) [30]. A comparison of the incidence rate reported in the study with that reported to the National Epidemiologic Surveillance program among similar pediatric populations in Managua revealed that there were 14 to 28 (average 21.3) times more dengue cases in the study each year per 100,000 persons than those reported to the surveillance program [31].

In a study undertaken in* Panama*, 457 confirmed cases of dengue were identified between 2000 and 2005, a period which included two epidemics (2001 and 2005). Of these cases, 57.6% were females, with an average age of 13 years, and 53% of cases reported having contact with other infected subjects in the 15 days prior to developing the disease [33]. Four of seven patients with dengue hemorrhagic fever died.

### 3.3. Andean Region

There were 15 studies identified for the Andean region and included data from Bolivia [34]; Ecuador [34]; Peru [34–44]; and Venezuela [45–48]. Specific data from Colombia were either included as part of a wider review of dengue in the Americas [16] or lacking.

In the Andean region, all dengue virus serotypes were reported to circulate [34]; DENV-3 predominated from 1999 to 2010 [35, 39–42], peaking in 2008 where it accounted for 46.6% of dengue cases [35]. DENV-2 predominated in 2011 (41% of all dengue cases analyzed) [36] and cocirculated mainly with DENV-1 [40, 41]. The highest incidence of DENV-4 occurred between 2006 and 2011, with a 53% overall seroconversion to DENV-4 among 2,997 participants of a cohort study during this period [42].

In* Peru*, an outbreak in 2001 resulted in 137 confirmed cases in the province of Trujillo, with the highest percentages of cases in Trujillo district (38.7%), and among 15–44-year-olds [37]. Other studies found that the risk for severe dengue was increased in patients under the age of 15 years, those with a history of dengue [35], and those needing repeated treatment for recurrent dengue symptoms [44], demonstrating that dengue was more common and generally more severe in young people [36].

A number of longitudinal dengue serological studies have been performed in the Amazonian city of Iquitos, Peru, since 1999 [39–42]. The school-based absenteeism active surveillance for febrile illness found that at baseline 80% of the study population were dengue seropositive, and that seroprevalence increased with age but with significant neighborhood variation in age-adjusted rates ranging from 67.1 to 89.9% [40]. During the first 15 months of the study (starting October 1999), when DENV-1 and DENV-2 cocirculated, incidence rates ranged from 2 to 3 infections/100 person-years. However, the introduction of DENV-3 during the second half of 2001 had three distinct periods: amplification over 5-6 months, replacement of previously circulating serotypes, and epidemic transmission peaking at an incidence of 89 infections/100 person-years [40]. A spatial analysis using acute case and seroconversion data obtained between 1999 and 2003 showed that the seroprevalence of previously circulating dengue serotypes could be a predictor of transmission risk for a different serotype—that is, clusters of DENV-1 and DENV-2 infections were mainly in the area of the city where mosquito density and previous dengue infection were both high [41]. Nonetheless, human movement appeared to be an underlying factor characterizing the spatial dimensions of dengue transmission.

In a comparison of the school-based absenteeism-based active surveillance with community-based (door-to-door) active surveillance of febrile cases during 2004, a higher number of febrile cases were detected in general (4.52/100 versus 1.64/100 person-years) and for dengue cases specifically (2.35/100 versus 1.29/100 person-years) in school-aged children through community-based rather than school absenteeism-based surveillance [39]. Subsequently, from September 2006 through February 2011, when serotypes 3 and 4 circulated, door-to-door surveillance for acute febrile illness among susceptible participants showed that 39% (420/1077) and 53% (1595/2997) had seroconverted to serotypes 3 and 4, respectively [42]. Symptomatic infection was detected in 7% and 10% of serotype 3 and 4 infections, and disease during postsecondary infections was reduced by 93% and 64% for the two serotypes, respectively, compared with primary and secondary infections. Although the disease rates with serotypes 3 and 4 were low, they constituted a significant proportion of apparent postsecondary infections (14% and 45%, resp.). In a seroepidemiologic study following an outbreak in Casma district in 2002, epidemiologic surveillance was found to have detected only 21% of the infected total estimated and 35.2% of symptomatic cases [38].

An outpatient passive surveillance study assessing the prevalence of nonhemorrhagic clinical manifestations of dengue by serotype between 2005 and 2010 in* Peru*,* Bolivia*,* Ecuador*, and* Paraguay* found that individuals with serotype 3 had a higher prevalence of musculoskeletal and gastrointestinal manifestations, and those with DENV-4 had a higher prevalence of respiratory and cutaneous manifestations [34].

The association between dengue, demographic, and climate factors across geographic regions of Peru was assessed between 1994 and 2008 [43]. Dengue was shown to be persistent in jungle areas with epidemics frequently occurring around March during the wettest months. Moreover, dengue appeared to be frequently imported into coastal regions from endemic jungle areas as well as from cities of other neighboring endemic countries, where conditions sustained year-round mosquito breeding.

*Venezuela* has experienced cyclical epidemics of DHF since 1989. In a study in the western region of the country in 2001, among children up to 12 years admitted to hospital with DHF, there was a slight predominance of 8–12-year-olds compared to the younger age groups [48]; 60% of those children admitted came from urban areas. A study conducted in the Municipality of Fernandez Feo (Tachira State) in 2003 found that most cases of dengue (65/193, 33.7%) occurred in adults aged 20–39 years; the highest incidence of dengue (24.7%) was found in San Rafael de El Pinal (capital city) and the highest incidence of DHF (22.2%) in Barrio Buenos Aires (a suburban population) [46]. In another study conducted in the west of the country, in the community of Churuguara in 2006, 46% of participants were found to be seropositive for dengue [47]. In a cohort study from 2006 to 2010 in Naguanagua, in the central region of the country, in which 1,216 dengue patients were identified, the age groups that accounted for most cases of dengue were children aged 5–9 years (303 cases = 24.9%) and 10–14 years (270 cases = 22.2%) [45].

### 3.4. Southern Cone

Seven studies were identified for the Southern Cone region and included data from Argentina [49–51]; Chile [52]; and Paraguay [34, 53]. Specific data from Uruguay were either included as part of a wider review of dengue in the Americas [16] or lacking. This region is characterized by the circulation of serotypes DENV-1 and DENV-3 [49, 52], although there are records of all serotypes being isolated in the region [34].

In* Argentina*, there was an outbreak in 2004 that lasted for 109 days in the province of Salta, with the highest incidence in the city of Tartagal [49]. A spatiotemporal analysis of clustering of 487 suspected cases showed outbreak centers and spreading patterns that were related to entomologic and epidemiologic factors. In another outbreak that occurred in the metropolitan area of Buenos Aires in 2009, 54.5% of the 227 confirmed dengue cases were believed to have been imported from the Bolivian Republic and the northern provinces of Argentina, and the rest were autochthonous [50]. In the same year overall, more than 26,000 infections and six deaths were declared by the ministry of health across the country (that year there were only 744 confirmed cases and 5 dengue-related deaths reported to PAHO, Table 2), but the number of infections could have been at least double those officially declared [51].

The only published report during the review period of an outbreak in* Chile* occurred on Easter Island in 2002, resulting in 636 cases of dengue [52]. The outbreak appeared to be caused by DENV-1. No cases of DHF were diagnosed. It was presumed that the source of the virus was tourists from either Brazil or Tahiti—most of the tourists were from Brazil; and although a much lower proportion came from the Pacific Islands, the same serotype had been circulating there at the time.

*Paraguay* had an outbreak with 1,884 confirmed cases in 2006 (822 confirmed cases reported to PAHO that year, Table 1). This included 55 cases of DHF, recorded for the first time in the country. A seroprevalence study one year later found DENV IgM seropositivity of 28% in a population of 47 children and adolescents (28 of whom were girls) [53].

### 3.5. Hispanic Caribbean Islands

Fourteen studies were identified for the Hispanic Caribbean island and included data from Cuba [54], the Dominican Republic [55, 56], and Puerto Rico [57–67]. In the Hispanic Caribbean, circulation of all four serotypes was reported in 2002, 2007, and 2010 [56, 57, 59]; DENV-1 occurred mainly in children and young adults aged 5–24 years in 2007 [59] and circulated together with DENV-4 between 2003 and 2004 [55]; DENV-2 was the most persistent serotype throughout the review period [55, 58–61, 63, 66].

DENV-2 has circulated continuously for 25 years in* Puerto Rico*, but the period from 1999 to 2003 was one of historically low DENV-2 circulation rates [64]. DENV-3 emerged in 1998 after a 21-year absence, followed by a period of rapid expansion which correlated with the gradual withdrawal of the other serotypes over seven years, before declining in 2008 to low or undetectable levels [65]. The factors underlying the expansion and collapse of DENV-3 were attributed to high virus genetic diversity and a large dengue-naïve population.

A postmortem analysis found high dengue seroprevalence [67]: antidengue IgM positivity was found in sera from 3% (23/780) and antidengue IgG positivity in 77% (597/777) of postmortems undertaken during December 2000, April 2001, and October 2001. In 2006, the predominant serotypes identified among 300 randomly selected adult blood donors (mean age 44.6 years), of whom 92% were dengue seropositive, were DENV-2 and DENV-3 (63%) [66]. A clinic-based enhanced surveillance system for dengue undertaken from June 2005 to May 2006 in single municipality recorded a seropositivity rate of 7.7 per 1,000 inhabitants, with the highest rate among 10–19-year-olds (13.4 per 1,000) [61]. Of the 156 seropositive cases identified 3 (1.9%) had DHF and 30 (19.2%) had at least one severe clinical manifestation. The majority of cases for which acute and convalescence samples were collected in the correct time scale were found to be second infections (77/105; 73%).

Following an island-wide dengue outbreak into 2007, DENV-3 (1,342, 61.7%) and DENV-2 (677, 31.1%) were the most often detected serotypes, and the incidence of laboratory-positive dengue was highest among those aged 10–14 years (19.0 per 10,000), followed by 15–19-year-olds (17.9 per 10,000) and infants (10.9 per 10,000) [59]. Of 40 patients who died of suspected dengue during the island-wide outbreak, 11 had a seropositive laboratory test result, but none of these deaths had been managed according to current WHO guidelines [60]. Incidentally, the total number of dengue-related deaths reported to PAHO for that year for Puerto Rico was only 9 (Table 2), much lower than in this single study. An analysis of 15,350 blood donations made during the outbreak recorded viremia rates of 1 per 529 (0.19%) samples [63]. In 12 samples, viral titers ranging from 10^5^ to 10^9^ copies/mL for DENV-1, DENV-2, and DENV-3 were detected by RT-PCR, all of which were infectious in mosquito culture. Of note, one recipient of a blood donation containing 10^8^ copies/mL of DENV-2 developed DHF after transfusion.

A novel influenza A (H1N1) strain was detected in Puerto Rico in 2009 which coincided with an increased proportion of laboratory negative suspected dengue cases reported to the surveillance system [58]. A study that undertook enhanced surveillance of acute febrile illnesses in a tertiary care hospital, in Ponce, between 29 September and 18 December 2009, found that among 284 enrolled patients there were 31 dengue, 136 influenza, and 3 enterovirus cases confirmed. About half (48%) of the confirmed dengue cases met the clinical criteria for influenza. However, those with confirmed dengue were more likely to have hemorrhage (81% versus 26%), rash (39% versus 9%), and a positive tourniquet test (52% versus 18%) compared with those with influenza. The authors of the study concluded that complete blood count and tourniquet test may help differentiate dengue from other acute febrile illnesses.

In 2010, Puerto Rico experienced a prolonged dengue epidemic which resulted in the greatest number of cases (26,766 suspected cases) and deaths (148 fatalities) ever recorded [57] (though only 9,883 confirmed cases (Table 1) and a total of 33 deaths were reported to PAHO that year (Table 2)). Of 7,426 RT-PCR-positive specimens assessed, DENV-1 (69.0%) and DENV-4 (23.6%) were more frequently identified than DENV-2 (7.3%) and DENV-3 (<0.1%), which represents a reversal of the predominant serotypes observed in the 2007 epidemic. Adults accounted for 47.1% of all laboratory-positive cases, 49.7% dengue cases with warning signs, and 11.1% with severe dengue, and they accounted for nearly all fatal dengue cases (37/40; 92.5%). About a fifth of cases were primary DENV infections, and children aged 1–4 years were the only group with predominantly more primary infection than secondary. There were significantly more primary infections with DENV-1 (28.5%) than DENV-2 (6.8%) and DENV-4 (7.1%).

In the* Dominican Republic*, there were three major dengue outbreaks in 1998, 2000, and 2002 [55]. Dengue fever was the most common clinical presentation accounting for 75% of cases seen in clinics and DHF for 19%. At the time of the publication of the report in 2005 [55], seven provinces had dengue rates higher than 32 per 100,000 inhabitants. A study of 1,008 adults attending blood banks and 201 children aged less than 10 years visiting a hospital in Santo Domingo between June and July 2002 found that most adults (98%) and children (56%) were dengue seropositive [56]. Seropositivity among children increased with age: prevalence of seropositivity increased from 0–5% among those aged 1-2 years to 25–65% among those aged 3–6 years and 76–92% among those aged ≥7 years. The high seropositivity observed among infants (50%) was attributed to maternal antibodies.

An epidemic was reported in* Cuba* from July to December 2006, with a peak in cases occurring in the epidemiologic week 26 [54]. Most of the epidemic cases were female (9,277/15,215 dengue cases (no data on the number of dengue cases were reported to PAHO that year, Table 1)) and predominantly adults (85.7% diagnosed cases were 15 years or older) and occurred in the Municipalities of Morón, Ciego de Ávila, Venezuela, and Baraguá, areas considered at high risk of dengue [54].

### 3.6. English-Speaking Caribbean Islands

Eight studies were identified for the English-speaking Caribbean Islands and included data from Barbados [68]; Jamaica [69]; Trinidad and Tobago [70–73]; and US Virgin Islands [74, 75]. Specific data from the other English-speaking Caribbean Islands (Anguilla, Antigua and Barbuda, Bermuda, British Virgin Islands, the Bahamas, Cayman Islands, Dominica, Grenada, Montserrat, Saint Kitts and Nevis, Saint Lucia, Saint Vincent and the Grenadines, and Turks and Caicos Islands) were either included as part of a wider review of dengue in the Americas [16] or lacking.

In* Barbados*, a population-based, retrospective study of all children up to the age of 16 years who presented over a 10-year period (January 2000 to December 2009) with febrile illness and suspected dengue infection (*n* = 1,809) was undertaken to assess the epidemiology, clinical presentation, immunological characteristics, morbidity, and mortality associated with dengue [68]. During the study period, the annual incidence of dengue ranged from 0.29 to 2.92 cases per 1,000 children, with most cases occurring between October and January. Most children presented with undifferentiated fever (287/545, 53%), followed by dengue fever (225/545, 41%), DHF (15/545, 3%), and dengue syndrome (18/545, 3%). Most dengue cases (73% of 213 laboratory-confirmed dengue cases) occurred as second infections, with 30% diagnosed among hospitalized children and an overall crude mortality rate of 0.3%.

In* Jamaica* [69], a seroprevalence study of the healthy population (*n* = 277) undertaken in 2009 found that all participants were dengue IgG seropositive and 3.6% (10/277) were dengue IgM seropositive. A significant association was found between dengue IgM seropositivity and gender (males 10/105 [9.5%] versus females 0/172 [0%]). The high dengue IgG seropositivity among the healthy population limits its usefulness as a dengue diagnostic test on this island.

In the* United States Virgin Islands*, the largest outbreak ever recorded occurred in 2005 with 331 suspected dengue cases representing 62.2 cases per 10,000 inhabitants [74]. Of these cases, 54% were hospitalized, 21% had hemorrhagic manifestations, 28% had thrombocytopenia, 5% had DHF, and one patient died. Among the 89 laboratory-positive hospitalized patients identified there were 15 (17%) who met the WHO criteria for DHF. Age was the only factor significantly associated with DHF. Subsequently in 2012, 27 suspected cases were reported by a school nurse in St. Croix among 369 students and staff members, which suggested that there may have been a larger island-wide dengue outbreak [75]. A follow-up retrospective case study of suspected dengue cases at St. Croix's only hospital looking for patients tested for anti-DENV IgM during that year found that 31% (61/194) of IgM tests done were seropositive, but of these only 22% (42/194) were reported to Virgin Islands Department of Health [75].

In* Trinidad* (no studies were identified that specifically included Tobago), a population-based study of the effects of climate and mosquito indices on the incidences of dengue undertaken between January 2002 and December 2004 reported that the incidence of DF in 2002 was 5.05 cases per 1000 inhabitants (due to a major outbreak) but declined to 0.49 case per 1000 in 2004 [72]. Monthly* Aedes aegypti* indices did not decline over the study period, suggesting that the decline in dengue incidence was due to the development of herd immunity. Although rainfall was significantly associated with dengue incidence, temperature was not. Indeed, dengue transmission in Trinidad was shown to occur at a variable level based on factors including seroprevalence, mosquito density, and climate [73]. Moreover, the mosquito density required for DF transmission may be high for Trinidad given the high seroprevalence rates. In a cross-sectional seroprevalence study of 125 cord blood samples collected between September 2003 and January 2004 [71], 94.4% of samples assessed were dengue seropositive.

A retrospective analysis of adult admissions at a tertiary hospital in Trinidad treated for dengue between 1 January and 31 December 2008 identified 186 dengue patients (overall across Trinidad and Tobago, 206 confirmed cases were reported to PAHO that year, Table 1) [70]. Of these patients, nearly all (184; 99%) had thrombocytopenia (45.2% had severe thrombocytopenia), 14 had hemorrhage (all minor except for 1 major hemorrhage case), 13 received platelet transfusion, and in 6 cases who received platelet transfusion there was no evidence of plasma leakage. Overall, 3.8% of patients met the WHO criteria for DHF or dengue shock syndrome, and no deaths were reported [70]. The two age groups with the highest dengue frequencies were adults aged 46–60 years (28.5%) and those aged 18–25 years (21.5%).

## 4. Discussion

It is generally accepted that dengue is a climate-sensitive disease. Local climate and the El Niño-Southern Oscillation (ENSO)—a fluctuation between unusually warm (El Niño) and cold (La Niña) sea surface temperatures in the tropical Pacific Ocean—are potentially important drivers of the interannual variability in dengue transmission. El Niño and La Niña events typically recur every 2–7 years and develop in association with large-scale atmospheric pressure oscillations. ENSO may be linked to local climate anomalies in certain regions of the world, therefore influencing the availability of mosquito larval habitat, larval development, adult biting activity, gonotrophic cycle, and viral replication in the mosquito [22, 24, 76, 77]. However, the actual influence of ENSO and local climate on dengue transmission remains controversial, with studies reporting inconsistent interannual associations. Dengue transmission in Mexico has been shown to be strongly associated with ENSO and minimum temperature, although not with precipitation [13, 78]. Likewise, DHF epidemics in Colombia, Suriname, and French Guiana have been associated with El Niño events, although the effects of El Niño on local climate varied by region [79]. In contrast, other studies undertaken in Mexico and Puerto Rico have found that ENSO and local climate were not important determinants of interannual variability in dengue incidence [80, 81]. Indeed the relationship between climate variables and vector-related factors that influence dengue transmission are probably complex. A systematic review and meta-analysis assessing the risk of dengue risk with temperature change that included 33 studies suggested that 22–29°C might be the critical temperature range for epidemic dengue transmission in endemic regions [82].

Since 2009, the WHO/PAHO criteria for probable dengue, laboratory-confirmed dengue, dengue with or without warning signs, and severe dengue have been progressively incorporated into the surveillance programs of countries in the region. Nevertheless, globally, dengue disease surveillance has been hampered by large differences between reported and estimated cases because of the variable quality of available data [83]. In this review there were significant gaps between surveillance data reported to the PAHO and those identified in the published studies where comparable data were available; there were generally fewer confirmed cases and deaths reported to PAHO than in the published studies. It is well recognized that there is a tendency for passive national surveillance systems to underreport dengue cases. Estimates of the number of dengue cases reported by national surveillance systems in Latin America may be up to 28-fold lower than the number of actual cases [31, 84–86], with a greater tendency for underreporting in adults than children [85].

Other variables that complicate the interpretation of regional surveillance data include the following: (i) differences in laboratory confirmation rates, with a limited confirmation of cases due to cost, requirement for technical expertise, and wide variability in assay sensitivity and specificity; (ii) lack of studies aimed at defining dynamics in health-seeking behavior; and (iii) changes in case definitions and classifications that further complicate the interpretation of surveillance data collected over time [13–15]. In addition, overlapping clinical features with other diseases such as Zika and Chikungunya, as well as the possible cross-reactivity between dengue and Zika and other flaviviruses when immunological methods are used for IgM detection, further hamper the reliable diagnosis of the illness in areas with active cocirculation of these viruses. The development and implementation of a generic protocol for the integrated surveillance of dengue is required to help standardize data collection among the different countries and to improve understanding and characterization of the disease. This will help strengthen decisions on vector control and disease prevention [87] and inform future vaccination strategies [88] in Latin America and the Caribbean [89].

While dengue affects all age groups, available data on burden or severity of dengue by age group in Latin America and English-speaking and Hispanic Caribbean Islands are inconsistent, with some studies suggesting a greater burden or severity of disease among children [25, 35, 36, 44] and others suggesting the burden or severity of disease to be the same or higher among older children (aged ≥ 15 years) and adults [37, 57]. In Southeast Asia, where dengue has been circulating for much longer, there is evidence of an increase in the incidence of dengue towards older age groups [90] and that this age shift has led to dengue primarily affecting the adult population in some countries [91]. A previous systematic review of the epidemiology and burden of dengue in Latin America and the Caribbean reported that adults aged 15 to 59 years were the age group most affected [92].

Most countries exhibit seasonality in dengue incidence pattern related to rainy and warmer seasons. Most cases are reported in the second half of the year in countries located on the Northern Hemisphere, whereas below the equator cases these mostly occur in the first half of the year. In countries with stable tropical conditions, such as Venezuela, cases may be reported throughout the year, with an increase during the rainy season [16]. The epidemiology of the disease among the countries studied may also reflect the diverse demographic, socioeconomic, geographic, and cultural peculiarities of the populations in this vast region of the world. Other potential nonclimate drivers include intrinsic factors (e.g., introduction of new serotypes, herd immunity, and strain-cross immunity) and other social-ecological drivers influencing vector populations and human exposure, such as vector control interventions, changes in urban poverty and infrastructure, land use change, and human movement [93, 94]. For example, in Costa Rica, the inverse relationship between dengue incidence and percentage of households with water supply may merely reflect the need for households without water supply to store water in containers, thus providing a potential habitat for mosquitos even during the dry season [22]. In Venezuela, prospective studies indicate a high cumulative incidence of DENV infections among 5–13-year-old school children in the central part of the country, suggesting that transmission occurs mainly at home in that region. The combinations of increasingly crowded living conditions, growing population density, precarious homes, and water storage issues caused by enduring problems in public services in large urban centers are the most likely factors that contribute to permanent dengue transmission and failure of vector control programs [95]. Similar results were found in the literature reviews undertaken for Brazil, Colombia, and Mexico [13–15].

Although the PAHO has provided standardized dengue case definitions based on the 1997 WHO publication, later revised in 2009 according to disease severity, each country has since adapted these definitions in accordance with their national experience leading to some inconsistencies in case definitions between countries. For instance, in some countries, such as Cuba, all notified cases are laboratory-confirmed, whereas in most other countries the criteria for reporting, even in nonepidemic circumstances, are by epidemiological association (where suspected cases are reported as confirmed when the virus is known to be circulating) with a variable fraction of cases laboratory-confirmed. In a few countries where surveillance data are reported as a combination of laboratory-confirmed cases and through epidemiological association, then the combined data can sometimes be disaggregated to ascertain the number laboratory-confirmed cases and the number of cases reported through epidemiological association (official case definition) [93].

It is clear that new approaches for measuring the relationship between case counts collected during passive surveillance and the actual number of cases contributing to transmission, including laboratory-confirmed, asymptomatic, and nonsevere, are needed in most Latin American countries. Moreover, the lack of coordination between surveillance and response to disease management during epidemics needs to be improved since even when the information is available, dengue control measures are often not implemented, or when implemented, they may not be executed thoroughly or correctly such as reported in Mexico and Colombia [13, 15].

## 5. Conclusions

All four dengue serotypes regularly circulate in the region, sometimes with a hyperendemic pattern (cocirculation of 2 or more serotypes) and have contributed to an increase in the number of outbreaks and populations affected across the region in recent years. In 2013, the last analyzed year in our review, cocirculation of all serotypes was reported in Guatemala, Nicaragua, Mexico, Martinique, Guadeloupe, Colombia, Venezuela, French Guiana, Peru, Brazil, and Argentina. Nonetheless, there appears to be a significant underreporting of dengue to the PAHO, which hinders assessment of the true burden of the disease across the region. In order to assess the impact of a new dengue vaccine that has recently been approved in a number of countries in the region, health authorities need to further improve their dengue surveillance systems using the WHO 2009 case definition, with better diagnosis algorithms, including differentiation from Zika, Chikungunya, and other flaviviruses. Although the benefits of creating early warning systems based on combining climatic, environmental, and host and vector-based data to forecast outbreaks are attractive, robust quantifiable associations between vector indices and dengue transmission are needed for reliable outbreak prediction modeling.

## Supplementary Material

Supplementary Table S1 is a summary of the information extracted and includes internal identification numbers of the articles, first author, citation details, the database where the item was obtained, country, region or city where the study was conducted, age and gender of the participants, number of dengue cases (DF and/or DHF), type of evidence where data was obtained (clinical study, case reports, etc.), study design, date(s) the study was undertaken, identified serotypes and method used for their identification. No attempt was made to contact authors for further clarification or missing information.

## Figures and Tables

**(a):** 

Year	2000			2001			2002			2003			2004			2005			2006		
Country	D & DHF^a^	TI^b^	Ser^c^	D & DHF^a^	TI^b^	Ser^c^	D & DHF^a^	TI^b^	Ser^c^	D & DHF^a^	TI^b^	Ser^c^	D & DHF^a^	TI^b^	Ser^c^	D & DHF^a^	TI^b^	Ser^c^	D & DHF^a^	TI^b^	Ser^c^
Antigua and Barbuda	8	11.94	4	20	30.77	3	5	7.69	3	1	1.54	ND	0	0.00	ND	0	0.00	ND	ND	0.00	ND
Argentina	1,700	4.59	ND	11	0.03	*∗*	214	0.57	1, 3	135	0.36	1, 2, 3	1,516	4.05	3	34	0.09	2	64	0.17	2, 3
Aruba	76	77.55	ND	0	ND	ND	25	ND	ND	ND	0.00	ND	4	3.85	3	ND	0.00	ND	ND	0.00	2, 3
Bahamas	0	0.00	ND	0	ND	ND	0	0.00	ND	0	0.00	2, 3	1	0.32	ND	0	0.00	ND	ND	0.00	ND
Barbados	744	275.56	1, 3	1,043	389.18	1, 2, 3, 4	740	276.12	3	22	8.21	1, 3	19	7.09	3	ND	0.00	1, 3	ND	0.00	ND
Belize	4	1.66	ND	3	1.30	ND	41	16.40	2	0	0.00	ND	34	14.72	3, 4	ND	0.00	1, 2, 3	ND	0.00	ND
Bolivia	73	0.88	1, 2	176	14.67	1	892	74.33	1, 2	1,002	50.10	1, 2, 3	682	34.10	1, 2, 3	618	30.90	2, 3	570	28.50	2, 3
Chile	ND		ND	ND		ND	636	ND	1	0	0.00	ND	0	0.00	ND	ND	0.00	ND	ND	0.00	ND
Costa Rica	4,907	434.63	1, 3	9,237	818.16	2	12,251	314.53	1, 2	264	8.14	1, 2	ND	0.00	1, 2	ND	0.00	1	ND	0.00	1, 2
Cuba	138	1.23	3, 4	11,432	101.58	3	3,011	26.75	ND	0	0.00	ND	0	0	ND	ND	0.00	ND	ND	0.00	ND
Curacao	10	4.61	3	0	0.00	ND	ND	0.00	ND	ND	0.00	ND	ND	0.00	ND	ND	0.00	ND	ND	0.00	ND
Dominica	15	21.13	3	5	7.04	3	0	0.00	ND	0	0.00	ND	0	0.00	ND	5	7.04	ND	ND	0.00	2, 4
Ecuador	22,937	181.38	1, 2, 3, 4	10,919	84.77	2, 3	5,833	45.29	2, 3	889	6.90	3	1,111	8.63	1, 3, 4	3,161	24.54	1, 3	1,539	11.48	1, 3
El Salvador	3,248	51.75	2	1,093	17.09	2	18,307	286.05	1, 2, 3, 4	3,782	59.12	2, 4	6,413	96.61	1, 2, 4	7,941	117.52	2, 4	8,428	123.90	1, 2, 4
Granada	27	29.03	2	12	12.77	2, 3	84	89.36	3	3	3.19	ND	0	0.00	ND	0	0.00	ND	ND	0.00	4
Guatemala	9,006	79.10	2	4,516	38.64	2, 4	7,599	65.02	2, 3, 4	996	8.52	1, 2, 3, 4	937	8.02	1, 2, 3, 4	767	6.56	1, 2, 3, 4	976	ND	1, 2, 4
Guyana	19	2.21	1, 2	60	7.86	2	202	26.47	3	4	0.52	ND	0	0.00	ND	47	6.16	ND	ND	0.00	ND
Haiti	ND		ND	ND		ND	ND		ND	ND		ND	ND		ND	1	0.32	ND	ND		ND
Honduras	13,642	210.36	2	9,077	138.05	ND	32,269	490.78	2, 3, 4	184	2.80	2, 4	ND	0.00	1, 2, 4	ND	0.00	1, 2, 4	ND	0.00	ND
Cayman Islands	0	0.00	ND	0	0.00	ND	1	2.50	ND	1	2.50	ND	0	0.00	ND	0	0.00	ND	ND	0.00	ND
British Virgin Islands	3	14.29	2, 3	23	95.83	2, 3	0	0.00	ND	0	0.00	ND	0	0.00	ND	1	4.17	ND	ND	0.00	ND
Jamaica	25	0.97	ND	39	1.50	ND	90	3.46	ND	45	1.73	ND	19	0.73	ND	9	0.35	ND	ND	0.00	ND
Nicaragua	7,317	144.21	2, 4	2,104	40.40	2, 3	2,157	41.42	1, 2, 4	2,799	53.74	1	343	6.59	1, 2, 4	ND	0.00	1, 2, 4	ND	0.00	1, 2, 4
Panama	317	11.10	1, 2, 3, 4	1,545	53.29	2	711	24.53	2	39	1.35	2	ND	0.00	1, 2, 3	2,000	68.99	1, 2	ND	0.00	ND
Paraguay	24,282	441.81	1	38	0.67	1, 2	1,871	33.20	1, 2, 3	137	2.43	3	12	0.21	3	36	0.64	2	822	14.58	3
Peru	5,486	21.38	1, 2, 4	23,329	89.41	1, 2, 3, 4	8,875	34.01	1, 3	340	1.30	1, 2, 3	ND	0.00	1, 2, 3	926	3.55	1, 2, 3, 4	1,209	4.63	3
Puerto Rico	2,433	62.88	1, 2, 3	5,233	132.41	2, 3	2,906	73.53	2, 3	1,328	33.60	1, 2, 3	735	18.60	2, 3, 4	ND	0.00	2, 3, 4	ND	0.00	1, 2, 3
Dominican Republic	3,462	40.75	1, 2, 3, 4	3,592	42.28	ND	3,194	37.60	2	1,053	12.40	2	833	9.31	2, 4	903	10.63	ND	1,887	22.21	1, 2
Saint Martin	ND		ND	ND		ND	ND		ND	ND		ND	ND		ND	ND		ND	ND		ND
Saint Vincent and the Grenadines	5	4.42	ND	3	2.63	3	125	109.65	3	3	2.63	3	1	0.88	ND	2	1.75	3	ND	0.00	ND
Saint Kitts and Nevis	5	12.82	2	89	234.21	2	20	52.63	2	2	5.26	ND	3	7.89	ND	0	0.00	ND	ND	0.00	ND
Saint Lucia	0	0.00	ND	292	195.97	3	51	34.23	3	0	0.00	ND	3	2.01	ND	1	0.67	4	ND	0.00	4
Surinam	1,073	257.31	1, 2	760	181.38	3	1,104	263.48	3	1	0.24	2	7	1.67	3	ND	0.00	1, 2, 3	ND	0.00	2
Trinidad and Tobago	2,066	159.54	1, 2, 4	2,244	172.62	2, 3	6,246	480.46	2, 3	121	9.31	3	11	0.85	ND	ND	0.00	3	ND	0.00	2, 3
Uruguay	ND		ND	ND		ND	ND		ND	0	0.00	ND	0	0.00	ND	0	0.00	ND	0	0.00	ND
Venezuela	21,101	87.30	1, 2, 3, 4	83,180	337.69	1, 2, 3, 4	37,676	152.96	2, 3, 4	ND	0.00	1, 2, 3	ND	0.00	1, 2, 3, 4	ND	0.00	1, 2, 3, 4	5,242	19.73	1, 2, 3, 4

Total	124,129	NA	NA	170,075	NA	NA	147,136	NA	NA	13,151	NA	NA	12,684	NA	NA	16,452	NA	NA	20,737	NA	NA

NA, not applicable; ND, no data available.

^*∗*^All imported.

^a^Cases of dengue and dengue hemorrhagic fever.

^b^Incidence rate per 100,000 inhabitants.

^c^Circulating serotype.

*Note*. Epidemiologic week, to which information is available according to PAHO/WHO reports, is not the same for all countries.

Data available from “http://www.paho.org/hq/index.php?option=com_topics&view=article&id=1&Itemid=40734”.

**(b):** 

Year	2007			2008			2009			2010			2011			2012			2013		
Country	D & DHF^a^	TI^b^	Ser^c^	D & DHF^a^	TI^b^	Ser^c^	D & DHF^a^	TI^b^	Ser^c^	D & DHF^a^	TI^b^	Ser^c^	D & DHF^a^	TI^b^	Ser^c^	D & DHF^a^	TI^b^	Ser^c^	D & DHF^a^	TI^b^	Ser^c^
Antigua and Barbuda	ND	0.00	ND	17	26.15	2	0	0.00	ND	3	4.62	ND	3	3.37	4	10	11.24	1	3	3.37	1
Argentina	49	0.13	2, 3	3	0.01	1	744	1.99	1	1,185	3.16	1, 2, 4	213	0.53	1, 2	274	0.68	1, 2, 3	2,921	7.29	1, 2, 3, 4
Aruba	ND	0.00	ND	ND	0.00	ND	845	812.50	1, 3	613	589.42	1, 2, 3, 4	1,311	1,285.29	1,4	413	404.90	ND	125	122.55	4
Bahamas	ND	0.00	ND	1	0.32	ND	0	0.00	ND	8	2.60	1, 2	213	62.28	1	5	1.46	ND	2	0.58	ND
Barbados	ND	0.00	ND	1	0.37	ND	2	0.75	3	542	202.24	1, 2, 3, 4	179	65.81	1, 2, 4	434	159.56	1, 2, 4	1,122	412.50	1, 2, 4
Belize	ND	0.00	1	ND	0.00	ND	292	90.68	ND	692	214.91	ND	578	179.50	ND	1,948	604.97	ND	1,202	352.49	ND
Bolivia	2,142	107.10	2, 3	321	16.05	ND	7,421	76.29	1, 2, 3	775	7.97	1, 2	7,515	77.26	1, 2, 3	6,467	66.49	2	1,276	13.12	1, 2, 4
Chile	28	0.62	1	ND	0.00	1	14	0.31	1,4	0	0.00	ND	1	0.02	1	34	0.75	ND	39	0.86	ND
Costa Rica	ND	0.00	1, 2	ND	0.00	1, 2	0	0.00	1, 2	0	0.00	1, 2, 3	3,160	69.25	1, 2, 3	9,224	202.15	1, 2, 3	218	4.78	1, 2, 3
Cuba	ND	0.00	ND	ND	0.00	ND	70	0.62	ND	0	0.00	ND	0	0.00	ND	0	0.00	ND	0	0.00	ND
Curacao	ND	0.00	ND	ND	0.00	ND	0	0.00	ND	820	377.88	1, 2	869	400.46	2	203	142.96	1	82	57.75	ND
Dominica	ND	0.00	2, 4	20	28.17	2	2	2.82	3	140	197.18	1	40	56.34	1,4	29	40.85	1,4	56	78.87	1,4
Ecuador	3,375	25.17	1, 3, 4	244	1.82	1, 3	650	4.85	1,4	65,105	485.57	ND	2,989	22.29	1, 2, 4	140	1.04	1, 2, 4	0	0.00	1, 2, 4
El Salvador	6,181	96.62	1, 2	1,388	21.70	ND	7,461	116.63	1, 2, 3, 4	8,979	140.36	1, 2	7,469	116.76	1, 2, 3, 4	12,899	201.64	1, 2, 3	11,118	173.80	1, 2, 3
Granada	ND	0.00	4	6	6.38	ND	10	10.64	2	92	97.87	1, 2	87	92.55	1	75	68.18	ND	155	140.91	ND
Guatemala	580	4.96	1, 2, 4	397	3.40	1, 2	1,704	14.58	2, 4	3,331	28.50	1, 2, 3, 4	687	5.88	1, 2	2,066	17.68	1, 2, 3, 4	2,450	20.96	1, 2, 3, 4
Guyana	ND	0.00	2, 3	ND	0.00	ND	492	64.48	ND	1,468	192.40	1,4	1,093	143.25	ND	1,120	146.79	1, 2, 4	1120	146.79	2, 4
Haiti	ND		ND	ND		ND	0	ND	ND	0	ND	ND	0	0.00	ND	2	0.02	ND	0	0.00	ND
Honduras	2,128	28.23	1, 2, 4	ND	0.00	2, 4	0	0.00	1, 2	2,524	38.39	1, 2, 3, 4	0	0.00	1, 2	4	0.05	1, 2	591	6.91	2, 3
Cayman Islands	ND	0.00	2, 4	ND	0.00	2	0	0.00	ND	8	20.00	2	2	5.00	2	36	78.26	1,4	41	89.13	1,4
British Virgin Islands	ND	0.00	3	15	62.50	2, 4	3	12.50	2	9	37.50	ND	33	137.50	ND	214	891.67	ND	126	525.00	ND
Jamaica	ND	0.00	2, 4	ND	0.00	3	0	0.00	ND	375	14.43	2	408	15.70	ND	545	19.32	1	159	5.64	1
Nicaragua	1,132	21.74	1, 2, 3	1,424	27.34	1, 2, 3, 4	3,100	59.52	1, 2, 3	1,049	20.14	1, 2, 3	1,238	23.77	1, 3	5,542	106.41	1, 2, 3	8,957	171.99	1, 2, 3, 4
Panama	ND	0.00	3	1,230	42.43	3	4,299	148.29	1, 3	927	31.98	1, 3	3,171	89.15	1, 2, 3	723	20.33	1, 2, 3	1,165	32.75	1, 2, 3
Paraguay	6,137	108.89	3	8	0.14	ND	2,201	39.05	1, 3	3,529	54.70	1, 2, 3	6,750	104.63	1, 2	2,463	36.91	2, 4	12,432	186.30	1, 2, 4
Peru	1,801	6.90	1, 2, 3, 4	3,209	11.47	1, 3, 4	6,495	23.21	1, 2, 3, 4	10,565	35.80	1, 2, 3, 4	8,827	29.91	1, 2, 3, 4	15,858	53.73	1, 2, 3, 4	10,867	36.82	1, 2, 3, 4
Puerto Rico	3,111	78.72	1, 2, 3, 4	741	18.75	1, 2, 3	2,171	54.93	1, 2, 3, 4	9,883	250.08	1, 2, 4	1,495	37.83	1, 2, 4	5,652	151.69	1, 2, 3, 4	9,232	230.14	1, 2, 4
Dominican Republic	6,069	71.44	1, 2, 3, 4	2,038	23.99	ND	4,779	56.26	1, 2, 4	6,885	81.05	1, 2, 4	950	11.18	2	830	9.77	2	405	4.77	1, 2, 4
Saint Martin	ND		ND	ND		ND	305	854.58	1, 2, 4	507	1,420.57	1, 2	96	266.67	1, 2	129	350.54	2, 4	1,298	3,465.03	2, 4
Saint Vincent and the Grenadines	ND	0.00	ND	6	5.26	ND	3	2.63	ND	133	116.67	1, 2	47	44.34	ND	193	182.08	ND	248	233.96	ND
Saint Kitts and Nevis	ND	0.00	3	49	128.95	3	2	5.26	3	19	50.00	1, 2	43	113.16	1, 4	1	2.04	4	45	91.84	4
Saint Lucia	ND	0.00	2, 3, 4	64	42.95	2	3	2.01	3	37	24.83	1, 2, 4	585	358.90	1	33	20.25	ND	202	123.93	4
Surinam	ND	0.00	2	24	5.73	ND	28	6.83	4	113	27.56	1, 2, 4	145	33.49	2, 4	337	77.83	1, 2, 4	8	1.85	2
Trinidad and Tobago	ND	0.00	3	206	15.85	2, 3	24	1.85	2	0	0.00	2	1,243	91.53	1,4	0	0.00	ND	0	0.00	ND
Uruguay	ND	0.00	ND	ND	0.00	ND	0	0.00	ND	0	0.00	ND	0	0.00	ND	0	0.00	ND	0	0.00	ND
Venezuela	48,186	175.34	1, 2, 3, 4	11,234	40.22	1, 2, 3, 4	5,282	18.61	1, 2, 3, 4	0	0.00	1, 2, 3, 4	0	0.00	1, 2, 3, 4	0	0.00	1, 2, 3, 4	0	0.00	1, 2, 3, 4

Total	80,919	NA	NA	22,646	NA	NA	48,402	NA	NA	120,316	NA	NA	51,440	NA	NA	67,903	NA	NA	67,665	NA	NA

**(a):** 

Year	2000			2001			2002			2003			2004			2005			2006		
Country	DHF^a^ DSS^b^ SD^c^	Deaths	CFR	DHF^a^ DSS^b^ SD^c^	Deaths	CFR	DHF^a^ DSS^b^ SD^c^	Deaths	CFR	DHF^a^ DSS^b^ SD^c^	Deaths	CFR	DHF^a^ DSS^b^ SD^c^	Deaths	CFR	DHF^a^ DSS^b^ SD^c^	Deaths	CFR	DHF^a^ DSS^b^ SD^c^	Deaths	CFR
Antigua and Barbuda	0	0	ND	0	0	ND	0	ND	ND	ND	ND	ND	ND	ND	ND	ND	ND	ND	0	0	0
Argentina	0	0	ND	0	0	ND	0	0	ND	0	0	ND	ND	ND	ND	0	0	ND	0	0	0
Aruba	ND	0	ND	ND	ND	ND	ND	ND	ND	ND	ND	ND	ND	ND	ND	ND	ND	ND	0	0	0
Bahamas	0	0	ND	0	0	ND	0	ND	ND	0	0	ND	ND	ND	ND	ND	ND	ND	0	0	0
Barbados	0	0	ND	14	0	ND	0	ND	ND	0	0	ND	ND	ND	ND	ND	ND	ND	0	0	0
Belize	0	0	ND	ND	ND	ND	0	ND	ND	ND	ND	ND	0	ND	ND	0	0	ND	0	0	0
Bolivia	0	0	ND	0	0	ND	1	1	ND	47	6	12.77	25	0	0	10	0	0	1	1	100.00
Chile	ND	ND	ND	ND	ND	ND	0	0	ND	0	0	ND	0	0	ND	ND	ND	ND	0	0	ND
Costa Rica	4	0	ND	37	0	ND	27	0	ND	69	0	0	11	0	0	52	2	3.9	72	0	0
Cuba	0	0	ND	69	2	ND	12	1	ND	0	0	ND	0	0	ND	ND	ND	ND	ND	ND	ND
Curacao	0	0	ND	ND	ND	ND	ND	ND	ND	ND	ND	ND	ND	ND	ND	ND	ND	ND	0	0	0
Dominica	0	0	ND	0	0	ND	0	ND	ND	0	0	ND	ND	ND	ND	4	ND	0	0	0	0
Ecuador	3	1	ND	55	0	ND	158	0	ND	416	5	1.20	64	2	3.13	334	14	4.19	173	6	3.47
El Salvador	411	26	ND	54	4	ND	405	11	ND	138	8	5.80	154	1	0.7	207	0	0	245	4	1.6
Granada	0	0	ND	0	0	ND	3	ND	ND	0	0	ND	0	ND	ND	0	0	ND	0	0	0
Guatemala	42	9	ND	4	2	ND	47	6	ND	22	3	13.6	39	4	10.3	32	1	3.13	6	1	16.7
Guyana	0	0	ND	ND	ND	ND	2	ND	ND	ND	ND	ND	0	ND	ND	0	0	ND	0	0	0
Haiti	ND	ND	ND	ND	ND	ND	ND	ND	ND	ND	ND	ND	ND	ND	ND	ND	ND	ND	ND	ND	ND
Honduras	314	10	ND	431	0	ND	863	17	ND	458	11	2.40	2345	2	0.1	1795	6	0.3	636	0	0
Cayman Islands	0	0	ND	0	0	ND	0	ND	ND	0	0	ND	0	ND	ND	0	0	ND	0	0	0
British Virgin Islands	0	0	ND	ND	ND	ND	0	ND	ND	0	0	ND	0	ND	ND	0	0	ND	0	0	0
Jamaica	0	0	ND	0	0	ND	0	ND	ND	0	0	ND	ND	ND	ND	ND	ND	ND	0	0	0
Nicaragua	636	4	ND	458	21	ND	157	12	ND	235	4	1.70	93	2	2.15	177	12	6.78	52	1	1.92
Panama	3	0	ND	7	1	ND	5	0	ND	0	0	ND	4	2	50.00	2	1	50.00	7	1	14.3
Paraguay	0	0	ND	0	0	ND	0	0	ND	0	0	ND	0	0	ND	0	0	ND	0	0	0
Peru	0	0	ND	251	4	ND	13	1	ND	15	0	0	35	1	2.86	16	0	0	4	0	0
Puerto Rico	24	0	ND	36	4	ND	23	1	ND	5	0	0	11	3	27.3	19	7	36.8	7	3	42.9
Dominican Republic	58	6	ND	4	0	ND	76	14	ND	252	75	29.76	136	13	9.56	84	18	21.4	230	53	23
Saint Kitts and Nevis	0	0	ND	4	0	ND	0	ND	ND	ND	ND	ND	ND	ND	ND	ND	ND	ND	0	0	0
Saint Lucia	0	0	ND	0	0	ND	0	ND	ND	0	0	ND	0	ND	ND	0	0	ND	0	0	0
Saint Martin	ND	ND	ND	ND	ND	ND	ND	ND	ND	ND	ND	ND	ND	ND	ND	ND	ND	ND	ND	ND	ND
Saint Vincent and the Grenadines	0	0	ND	0	0	ND	2	ND	ND	0	0	ND	0	ND	ND	0	0	ND	0	0	0
Surinam	4	9	ND	12	0	ND	23	ND	ND	1	ND	0	7	ND	0	141	ND	0	32	0	0
Trinidad and Tobago	49	0	ND	86	0	ND	273	12	ND	80	ND	0	49	ND	0	0	0	ND	1	0	0
Uruguay	ND	ND	ND	ND	ND	ND	ND	ND	ND	0	0	ND	0	0	ND	0	0	ND	0	0	0
Venezuela	2186	5	ND	6541	15	ND	2979	1	ND	2246	7	0.31	1986	5	0.25	2681	4	0.15	2476	0	0

Total	3734	70	NA	8063	53	NA	5069	77	NA	3984	119	NA	4959	35	NA	5554	65	NA	3942	70	NA

CFR, case fatality rate; NA, not applicable; ND, no data available.

^a^Cases of dengue hemorrhagic fever.

^b^Cases of dengue hemorrhagic fever/dengue shock syndrome and complicated dengue cases.

^c^Cases of severe dengue and complicated dengue cases.

*Note*. Epidemiologic week, to which information is available according to PAHO/WHO reports, is not the same for all countries.

**(b):** 

Year	2007			2008			2009			2010			2011			2012			2013		
Country	DHF^a^ DSS^b^ SD^c^	Deaths	CFR	DHF^a^ DSS^b^ SD^c^	Deaths	CFR	DHF^a^ DSS^b^ SD^c^	Deaths	CFR	DHF^a^ DSS^b^ SD^c^	Deaths	CFR	DHF^a^ DSS^b^ SD^c^	Deaths	CFR	DHF^a^ DSS^b^ SD^c^	Deaths	CFR	DHF^a^ DSS^b^ SD^c^	Deaths	CFR
Antigua and Barbuda	0	0	0	0	0	0	0	0	0	0	0	0	0	0	0	0	0	0	0	0	0
Argentina	0	0	0	0	0	0	6	5	83.33	0	0	0	0	0	0	0	0	0	0	0	0
Aruba	0	0	0	0	0	0	2	0	0	3	1	33.33	2	2	0.07	0	0	0	1	1	0
Bahamas	0	0	0	0	0	0	0	0	0	0	0	0	3	0	0	0	0	0	0	0	0
Barbados	0	0	0	0	0	0	1	0	0	52	4	7.69	6	2	0.27	15	1	0.07	16	4	0
Belize	0	0	0	0	0	0	87	0	0	293	3	1	3	0	0	6	0	0	12	0	0
Bolivia	109	1	0.92	3	0	0	198	25	12.63	0	0	0	48	47	0.18	2011	37	0.09	63	8	0.06
Chile	0	0	0	0	0	0	0	0	0	0	0	0	0	0	0	0	0	0	0	0	0
Costa Rica	318	8	2.5	52	0	0	8	0	0	21	0	0	28	0	0	54	0	0	151	1	0
Cuba	ND	ND	ND	ND	ND	ND	0	0	ND	0	0	ND	0	0	ND	0	0	ND	0	0	ND
Curacao	ND	ND	0	ND	ND	0	0	0	0	1	1	100.00	2	2	0.13	0	0	0	0	0	0
Dominica	1	0	0	0	0	0	0	0	0	2	0	0	0	0	0	0	0	0	0	0	0
Ecuador	334	5	1.50	15	0	0	89	1	1.12	0	0	0	92	8	0.10	290	23	0.14	71	11	0.08
El Salvador	100	0	0	0	ND	0	112	10	8.9	185	2	1.1	213	7	0	778	6	0	425	3	0
Granada	0	0	0	0	0	0	0	0	0	7	0	0	5	0	0	0	0	0	0	0	0
Guatemala	21	4	19.1	3	0	0	238	28	11.8	203	41	20.20	12	9	0.35	43	17	0.18	33	8	0.07
Guyana	1	0	0	0	0	0	1	1	100.00	1	1	100.00	0	0	0	20	0	0	0	0	0
Haiti	ND	ND	ND	ND	ND	0	0	0	0	0	0	0	0	0	0	0	0	0	0	0	0
Honduras	4180	16	0.4	2481	9	0.4	763	14	1.8	3268	83	2.5	885	0	0	2730	4	0	4398	29	0.1
Cayman Islands	ND	ND	0	0	0	0	0	0	0	0	0	0	0	0	0	0	0	0	0	0	0
British Virgin Islands	0	0	0	0	0	0	0	0	0	0	0	0	0	0	0	0	0	0	0	0	0
Jamaica	0	0	ND	2	0	0	0	0	0	158	15	9.49	4	0	0	12	10	0.21	16	4	0.46
Nicaragua	151	12	7.95	34	5	14.7	80	11	13.8	104	3	2.88	7	1	0.01	47	5	0.02	151	20	0.03
Panama	3	0	0	3	0	0	28	6	21.4	1	0	0	38	17	0.44	6	0	0	12	8	0.25
Paraguay	55	17	30.91	ND	ND	0	0	0	0	30	15	50.00	98	62	0.14	119	70	0.18	2076	251	0.17
Peru	35	2	5.71	32	1	3.13	12	1	8.33	59	7	11.86	198	31	0.10	205	41	0.14	69	18	0.13
Puerto Rico	48	9	18.75	65	2	3.08	63	2	3.17	33	33	100.00	31	3	0.05	26	7	0.05	50	12	0.07
Dominican Republic	225	47	20.89	183	38	20.77	995	52	5.23	1025	51	4.98	110	2	0.09	141	71	0.73	424	111	0.67
Saint Kitts and Nevis	0	0	0	0	0	0	0	0	0	0	0	0	1	0	0	0	0	0	0	0	0
Saint Lucia	0	0	0	0	0	0	0	0	0	0	0	0	1	1	0.17	0	0	0	4	2	0.85
Saint Martin	ND	ND	ND	ND	ND	ND	0	1	0	12	0	0	0	0	0	2	0	0	31	2	0.06
Saint Vincent and the Grenadines	0	0	0	0	0	0	0	0	0	1	0	0	0	0	0	4	0	0	4	0	0
Surinam	0	0	0	12	0	0	69	0	0	20	1	5.00	24	8	1.96	182	4	0.60	0	0	0
Trinidad and Tobago	0	0	0	8	3	0	0	0	0	3	3	100.00	1	1	0.08	13	0	0	17	0	0
Uruguay	0	0	0	ND	ND	0	0	0	0	0	0	0	0	0	0	0	0	0	0	0	0
Venezuela	6461	ND	0	3649	0	0	5149	0	0	10203	0	0	1416	0	0	1931	0	0	583	0	0

Total	12042	121	NA	6542	58	NA	7901	157	NA	15685	264	NA	3228	203	NA	8635	296	NA	8607	493	NA
