# Supplementary material for: Epidemiological Characteristics of Dengue Disease in Latin America and in the Caribbean: A Systematic Review of the Literature
Source: J Trop Med. 2017 Mar 14;2017:8045435. doi: 10.1155/2017/8045435 (PMC5368385; doi:10.1155/2017/8045435)
Supplement: Supplementary file 1 — Supplementary Table S1 is a summary of the information extracted and includes internal identification numbers of the articles, first author, citation details, the database where the item was obtained, country, region or city where the study was conducted, age and gender of the participants, number of dengue cases (DF and/or DHF), type of evidence where data was obtained (clinical study, case reports, etc.), study design, date(s) the study was undertaken, identified serotypes and method used for their identification. No attempt was made to contact authors for further clarification or missing information. [file 8045435.f1.docx]

| **Table S1.** Articles selected for the systematic review of dengue in Latina America (n=60). | | | | | | | | |
| --- | --- | --- | --- | --- | --- | --- | --- | --- |
| **N°** | **Number in current manuscript reference list** | **ID** | **Citation** | **Databases** | **Country** | **Study design** | **Date of the study** | **Number of cases/ Sample studied** |
| 1 | 17 | 56 | Recio-Domingo M et al., 2002 | LILACS | Cantón de Esparza, Puntarenas, Costa Rica | Case series study | January 1997 to June 2002 | 1. 1997: 1,347 cases 2. 1998: 9 cases 3. 1999: 100 cases 4. 2000: 177 cases 5. 2001: 213 cases 6. 2002 (January-June): 195 cases |
| 2 | 52 | 31 | Perret C et al., 2003 | PUBMED | Villa Hanga Roa, Isla de Pascua, Chile | Case series study | 2002 | 1. March 2002: first confirmed case 2. Blood samples were taken from 16 febrile patients 3. A serum simple was taken from 423 asymptomatic convalescent patients 4. January to May 2002: Dengue Fever (DF) Outbreak, 636 dengue cases were diagnosed. |
| 3 | 54 | 28 | Posada-Fernández P et al., 2010 | LILACS | Ciego de Ávila Province, Cuba | Case series study | 2006 | 15415 febrile cases were identified |
| 4 | 55 | 42 | Cabrera-Batista B et al., 2005 | PUBMED | República Dominicana | Case series study | 1-43 week, 2003-2004 | 1. 1–43 week, 2003: 1686 cases 2. 1–43 week, 2004: 4712 cases |
| 5 | 57 | 118 | Sharp TM et al., 2013 | PUBMED | Puerto Rico | Case series study | 1^st^ January to 31^st^ December, 2010 | 26,766 suspected dengue cases were identified |
| 6 | 74 | 140 | Mohammed H et al., 2010 | PUBMED | St Croix, Islas Vírgenes US | Case series study | 1^st^ January to 31^st^ December, 2005 | 331 suspected dengue fever cases were reported |
| 7 | 73 | 175 | Chadee DD, 2009 | PUBMED | Country Victoria, Trinidad, West Indies | Case series study | June-November, 2004 | 50 confirmed/suspected cases of DF were investigated, and 35 were confirmed as DF/DHF cases |
| 8 | 18 | 51 | Ramírez-Salas A et al., 2009 | LILACS | San Jose, Costa Rica | Case series study | 2003-2007 | 1,479 suspected dengue cases, of which 688 were laboratory confirmed cases |
| 9 | 25 | 93 | Hammond SN et al., 2005 | PUBMED | Leon and Managua, Nicaragua | Case series study | January 1999 to December 2001 | 3,173 suspected dengue cases |
| 10 | 58 | 122 | Lorenzi OD et al., 2013 | PUBMED | Ponce, Puerto Rico | Case series study | 29^th^ September to 18^th^ December, 2009 | 284 patients |
| 11 | 19 | 48 | Alfaro-Obando A et al., 2006 | LILACS | Liberia (Canton), Costa Rica | Case series study | 2003 | 1,153 dengue cases were analyzed |
| 12 | 33 | 104 | Larrú-Martínez B et al., 2006 | PUBMED | Panama | Case series study | 1^st^ January 2000 to 30^th^ August 2005 | 457 cases were confirmed by laboratory |
| 13 | 34 | 45 | Halsey ES et al., 2012 | PUBMED | Peru (La Merced, Puerto Maldonado, Piura, Tumbes, Yurimaguas), Bolivia (Concepcion, Magdalena, Santa Cruz, Villa Tunari), Ecuador (Guayaquil, Puyo), and Paraguay (Asuncion, Central, Ciudad del Este, Encarnacion, Filadelfia) | Case series study | 1^st^ January 2005 to 20^th^ de August 2010 | 1,716 individuals were identified as infected with a DENV |
| 14 | 35 | 12 | Mamani E et al., 2010 | LILACS | Piura, Peru | Case series study | May-June, 2008 | 73 serum samples from patients with dengue were analyzed |
| 15 | 36 | 213 | Fiestas-Solórzano V et al., 2011 | PUBMED | Iquitos, Peru | Case series study | 23^rd^ January to 5^th^ February, 2011 | 41 hospitalized dengue patients were analyzed, of which 69% were classified as dengue cases with warning signs and 31% as severe dengue cases |
| 16 | 45 | 1 | Stranieri MM y Palacios M, 2013 | MedicLatina | Naguanagua, Venezuela | Case series study | 2006-2010 | 1,216 dengue patients were detected |
| 17 | 46 | 5 | Arria M et al., 2007 | Medigraphic | Estado de Tachira, Venezuela | Case series study | 2003 | 123 cases were reported (97 cases of dengue fever and 26 cases of dengue hemorrhagic) |
| 18 | 49 | 16 | Rotela C et al., 2007 | PUBMED | Tartagal, Argentina | Case series study | 24^th^ January to 11^th^ May, 2004 | 487 suspected dengue cases were recorder and geo-referenced |
| 19 | 50 | 9 | Seijo A et al., 2009 | LILACS | Buenos Aires Metropolitan Area | Case series study | January-May, 2009 | 1654 patients of which 227 were confirmed dengue cases |
| 20 | 60 | 127 | Tomashek KM et al., 2012 | PUBMED | Puerto Rico | Case series study | 2007 | 10,576 suspected dengue cases were reported |
| 21 | 70 | 174 | Sharma A et al., 2012 | PUBMED | Trinidad and Tobago | Case series study | 1^st^ January to 31^st^ December, 2008 | 186 patients |
| 22 | 68 | 193 | Kumar A et al, 2013 | PUBMED | Barbados | Cross-sectional study | January 2000 to December 2009 | 702 laboratory confirmed cases of dengue detected among the 1809 children with febrile illness |
| 23 | 71 | 179 | Campbell CA et al, 2007 | PUBMED | Trinidad y Tobago | Cross-sectional study | September 2003 to January 2004 | Cord blood samples from 125 births were collected at the two hospital sites |
| 24 | 61 | 151 | Ramos MM et al., 2008 | PUBMED | Sudeste de Puerto Rico | Cross-sectional study | June 2005 to May 2006 | 1,393 suspected dengue cases |
| 25 | 59 | 146 | Tomashek KM et al., 2009 | PUBMED | Puerto Rico | Cross-sectional study | 2007 | 10,508 suspected dengue cases, on average 202 suspected cases were reported per week |
| 26 | 62 | 152 | Mohammed H et al., 2008 | PUBMED | Puerto Rico | Cross-sectional study | 20^th^ September to 4^th^ December, 2005 | 16,521 blood donations were collected during the study period |
| 27 | 47 | 20 | Morillo EG, 2006 | LILACS | Estado de Falcon, Venezuela | Cross-sectional study | Mar-Aug, 2003 | 196 asymptomatic cases |
| 28 | 63 | 128 | Stramer SL et al., 2012 | PUBMED | Puerto Rico | Cross-sectional study | 1^st^ June to 31^st^ December, 2007 | 15,350 samples randomly selected from 28,227 samples |
| 29 | 64 | 139 | McElroy KL et al., 2011 | PUBMED | Puerto Rico | Cross-sectional study | 1986 to 2007 | Dengue cases ranged from 2,000 to ≈16,000 per year |
| 30 | 65 | 124 | Santiago GA et al., 2012 | PUBMED | Puerto Rico | Cross-sectional study | 1998 to 2007 | Genomes of 92 DENV-3 clinical isolated obtained from human sera |
| 31 | 20 | 53 | Iturrino-Monge R et al., 2006 | LILACS | Puntarenas (Costal Province) and San Jose (Capital City of Costa Rica) | Cross-sectional study | July 2002 to July 2003 | Blood samples were taken of 206 children (103 children in each region) |
| 32 | 23 | 57 | Avila-Montes GA et al., 2010 | LILACS | Honduras | Cross-sectional study | 1991-2010 | Epidemiologic statistics were reviewed about Dengue in Honduras, and it was found an increase of dengue cases with epidemic peaks each three or four years from 1991:   1. 3,045 cases (1991) 2. 18,152 cases (1995) 3. 21,359 cases (1998) 4. 32,269 cases (2002) 5. 29,328 cases (2007) 6. 63,477 cases (2010) |
| 33 | 37 | 14 | Otiniano-Oyola A y Uribe-Uribe LJ, 2002 | LILACS | Provincia de Trujillo, Peru | Cross-sectional study | 2001 | Until epidemiological week 22 (2001) were registered 137 confirmed dengue cases |
| 34 | 38 | 282 | Gómez B et al., 2005 | Redalyc | Casma, Ancash, Peru | Cross-sectional study | February to April, 2002 | 403 surveys and were collected 400 blood samples in 9 sectors of the District of Casma |
| 35 | 48 | 35 | González L et al., 2004 | LILACS | Ciudad San Cristobal, Estado de Tachira, Venezuela | Cross-sectional study | Jul-Sep, 2001 | 82 children admitted to the hospital as suspected dengue cases |
| 36 | 53 | 61 | Giménez V et al., 2011 | LILACS | Asuncion, Central, Cordillera, Guaira, Canindeyu, Caaguazu, Caazapa, Paraguari | Cross-sectional study | February-April, 2007 | 47 children and adolescents of which 13 were IgM positive for DENV |
| 37 | 56 | 44 | Yamashiro T et al., 2004 | PUBMED | Santo Domingo, Dominican Republic | Cross-sectional study | 1. Sample collection from adults: 17^th^ June to 23^th^ July, 2002. 2. Sample collection from children: 30^th^ July to 27^th^ August, 2002 | 1. Samples from adults: 1,008 adult samples. 2. Samples from children: 201 samples. |
| 38 | 66 | 131 | Mohammed H et al., 2012 | PUBMED | Puerto Rico | Cross-sectional study | 1^st^ February to 31^st^ March, 2006 | A simple of 300 blood donations |
| 39 | 67 | 77 | Rigau-Pérez J et al., 2006 | LILACS | Puerto Rico | Cross-sectional study | December 2000, April 2001, and October 2001 | 835 post-mortem examinations |
| 40 | 69 | 74 | Brown MG et al., 2009 | PUBMED | Jamaica | Cross-sectional study | NSR | 277 serum samples, 3.6% were positive for dengue IgM antibodies and dengue IgG antibodies were found in100% |
| 41 | 26 | 92 | Balmaseda A et al., 2006 | PUBMED | Managua, Nicaragua | Cohort Study | Annual blood samples were collected prior to the dengue  season in May of 2001, May of 2002, and March of 2003  for serological testing for anti-DENV antibodies. | 1) In 2001: 602 children  2) In 2002: 396 children were newly recruited prior  3) Paired annual samples for 2001–2002 and 2002–2003 were available from 467 (78%) and 719 (84%) participants, respectively. 3) 398 children remained enrolled for the entire duration of the study |
| 42 | 30 | 286 | Gordon A et al., 2013 | PUBMED | Managua, Nicaragua | Cohort study | August 2004 to June 2010 | During the first six years of the study, 5,545 children participated. Yearly participation ranged from 3,693 to 3,953 children:  1) 2004-2005: 3,721 participants, 2) 2005-2006: 3,695 participants, 3) 2006-2007: 3,795 participants, 4) 2007-2008: 3,693 participants, 5) 2008-2009: 3,953 participants, 6) 2009-2010: 3,969 participants |
| 43 | 31 | 84 | Standish K et al., 2010 | PUBMED | Managua, Nicaragua | Cohort study | 2004-2008 | 4,742 children participated  in the study, with a yearly active cohort of 3,693–3,795 children |
| 44 | 39 | 220 | Rocha C et al., 2009 | PUBMED | Iquitos, Peru | Cohort study | April to December,  2004 | In the 9 months that the two  programs were operated concurrently, were monitored  1,135 students (99.5%, 5–17 years of age) in the school-based  surveillance, and 4,850  residents (0–98 years of age) in the community-based surveillance |
| 45 | 27 | 285 | Montoya M et., 2013 | No dice en qué base de datos lo encontraron, pero lo encontré en PUBMED | Managua, Nicaragua | Cohort study | August 2004 to March 2011 | 5,541 children participated: 3,713 were enrolled at the onset of the study and 1,828 in subsequent years |
| 46 | 28 | 85 | Balmaseda A et al., 2010 | PUBMED | Managua, Nicaragua | Cohort study | August 2004 to July 2008 | The cohort population varied:   1. 2004–5: 3,713 2. 2005-6: 3,689 3. 2006-7: 3,563 4. 2007-8: 3,676 |
| 47 | 40 | 216 | Morrison AC et al., 2010 | PUBMED | Iquitos, Peru | Cohort study | 21^st^ February 1999  to 12^th^ February 2005 | To maintain  an active cohort of 2,400 study participants, a total of 4,586  participants were enrolled in the study |
| 48 | 41 | 210 | Liebman KA et al., 2012 | PUBMED | Iquitos, Peru | Cohort study | Six-month  intervals from 1999 to 2003 | 3,110 participants |
| 49 | 42 | 205 | Olkowski S et al., 2013 | PUBMED | Iquitos, Peru | Cohort study | September 2006 to February 2011 | 1. Cohort A: 2,356 participants 2. Cohort B: 2,445 participants |
| 50 | 29 | 81 | Gutierrez G et al., 2011 | PUBMED | Managua, Nicaragua | Cohort study | During dengue season: July 1–June 30 of each year in years 2005–2010 and August 1, 2004–June 30, 2005 in the first year of the study. | Between August 2009 and June 2010, 170 laboratory confirmed symptomatic cases of dengue were identified among 3,711 active cohort participants and in the hospital-based study, 212 dengue cases were confirmed among 396 study participants during August 2009- January 2010 |
| 51 | 16 | 64 | San Martín JL et al., 2010 | PUBMED | Las Américas | Ecologic study | Three periods:   1. 1980-1989 (80’s) 2. 1990-1999 (90’s) 3. 2000-2007 (2000-7) | Total dengue cases reported in the Region were:   1. 1,033,417 (80’s) 2. 2,725,405 (90’s) 3. 4,759,007 (2000-7) |
| 52 | 72 | 180 | Chadee DD, 2007 | PUBMED | Trinidad and Tobago | Ecologic study | January 2002 to December 2004 | 9,275 dengue cases were reported along the study period (3 years):   1. 2002: 6314 cases 2. 2003: 2340 cases 3. 2004: 621 cases |
| 53 | 43 | 211 | Chowell G et al., 2011 | PUBMED | Peru | Ecologic study | 1994 to 2008 | 86,631 dengue cases were reported |
| 54 | 21 | 47 | Wong-McClure R et al., 2007 | LILACS | Costa Rica | Ecologic study | 1999 to 2004 | 10,308 dengue cases were reported during the study period, 2003 was the year with the highest weekly average of dengue cases (43) |
| 55 | 22 | 32 | Mena N et al., 2011 | PUBMED | Costa Rica | Ecologic study | 1999-2007 | It was not mentioned the number of cases, only that incidence of D/DH per canton was calculated from total accumulated of D/DH cases during 1999-2007, these data was obtained from annual cases recorded for each canton in the statistical office of the Ministry of Health (Costa Rica) |
| 56 | 24 | 284 | Zambrano LI et al., 2012 | PUBMED | Tegucigalpa, Honduras | Ecologic study | 1^st^ January- 31^st^ December, 2010 | 3,353 cases of DHF were diagnosed and reported |
| 57 | 44 | 4 | Súarez-Ognio L et al., 2011 | LILACS | Iquitos, Peru | Case-control study | October 2010 to February 2011 | 73 cases (with severe dengue) and 153 controls (102 with warning signs and 51 without warning signs) |
| 58 | 32 | 83 | Reyes M et al., 2010 | PUBMED | Managua, Nicaragua | Cluster Study | 16^th^ October 2006- 12^th^ January 2007 and 8^th^ September-29^th^ November, 2007 | 1. 875 initial medical consultations to the Health center Sócrates Flores Vivas (HCSFV) for possible DENV infections by cohort participants occurred, of which 13 in 2006 and 64 in 2007 were laboratory-confirmed dengue cases 2. A total of 22 index participants were selected from the cohort study during the peak of dengue transmission, 7 in 2006 season and 15 in the 2007 season 3. At each site, 18-25 contacts were enrolled, totaling 495 contacts |
| 59 | 51 | 68 | Bernardini Zambrini DA, 2011 | LILACS | Argentina | Expert opinion | 2009 | This study reflects on the impact of the 2009 dengue epidemic in Argentina with more than 26,000 infections and six official deaths |
| 60 | 75 | 121 | CDC, 2013 | PUBMED | St Croix, US Virgin Islands | Note to the editor | November 2012 | 27 suspected dengue cases |

DENV, dengue virus; DF, dengue fever; DHF, dengue hemorrhagic fever
